# Supplementary material for: Psychometric properties of novel instrument for evaluating ambient air pollution health literacy in adults
Source: PLoS One. 2023 Jun 16;18(6):e0285001. doi: 10.1371/journal.pone.0285001 (PMC10275446; doi:10.1371/journal.pone.0285001)
Supplement: S1 Table — (DOCX) [file pone.0285001.s001.docx]

**S1 Table. Results of statistical fit indices for the confirmatory factor analysis (CFA) for sex-stratified subgroup**

| Fit index | 4-factor model | 3-factor model | 12-factor model | Critical value |
| --- | --- | --- | --- | --- |
| Male (n=576) | |  |  |  |
| Absolute fit indices | |  |  |  |
| RMSEA | 0.094 | 0.093 | 0.077 | $\leq$ 0.08 |
| SRMR | 0.067 | 0.065 | 0.048 | $\leq$ 0.08 |
| Incremental fit indices | |  |  |  |
| CFI | 0.821 | 0.820 | 0.909 | $\geq$ 0.90 |
| NFI | 0.776 | 0.775 | 0.873 | $\geq$ 0.90 |
| TLI | 0.799 | 0.800 | 0.865 | $\geq$ 0.90 |
| Female (n=721) | |  |  |  |
| Absolute fit indices | |  |  |  |
| RMSEA | 0.093 | 0.096 | 0.043 | $\leq$ 0.08 |
| SRMR | 0.067 | 0.062 | 0.069 | $\leq$ 0.08 |
| Incremental fit indices | |  |  |  |
| CFI | 0.843 | 0.833 | 0.936 | $\geq$ 0.90 |
| NFI | 0.796 | 0.785 | 0.897 | $\geq$ 0.90 |
| TLI | 0.824 | 0.815 | 0.906 | $\geq$ 0.90 |
