# Supplementary material for: Psychometric properties of novel instrument for evaluating ambient air pollution health literacy in adults
Source: PLoS One. 2023 Jun 16;18(6):e0285001. doi: 10.1371/journal.pone.0285001 (PMC10275446; doi:10.1371/journal.pone.0285001)
Supplement: S2 Table — (DOCX) [file pone.0285001.s002.docx]

**S2 Table. Results of statistical fit indices for the CFA for age subgroup**

| Fit index | 4-factor model | 3-factor model | 12-factor model | Critical value |
| --- | --- | --- | --- | --- |
| Aged 20-64 (n=1157) | |  |  |  |
| Absolute fit indices | |  |  |  |
| RMSEA | 0.089 | 0.089 | 0.067 | $\leq$ 0.08 |
| SRMR | 0.062 | 0.059 | 0.039 | $\leq$ 0.08 |
| Incremental fit indices | |  |  |  |
| CFI | 0.845 | 0.841 | 0.934 | $\geq$ 0.90 |
| NFI | 0.819 | 0.815 | 0.912 | $\geq$ 0.90 |
| TLI | 0.826 | 0.824 | 0.903 | $\geq$ 0.90 |
| Aged 65 or over (n=140) | |  |  |  |
| Absolute fit indices | |  |  |  |
| RMSEA | 0.144 | 0.156 | 0.122 | $\leq$ 0.08 |
| SRMR | 0.111 | 0.107 | 0.099 | $\leq$ 0.08 |
| Incremental fit indices | |  |  |  |
| CFI | 0.717 | 0.661 | 0.844 | $\geq$ 0.90 |
| NFI | 0.569 | 0.524 | 0.718 | $\geq$ 0.90 |
| TLI | 0.682 | 0.625 | 0.769 | $\geq$ 0.90 |
