# Supplementary material for: Psychometric properties of novel instrument for evaluating ambient air pollution health literacy in adults
Source: PLoS One. 2023 Jun 16;18(6):e0285001. doi: 10.1371/journal.pone.0285001 (PMC10275446; doi:10.1371/journal.pone.0285001)
Supplement: S3 Table — (DOCX) [file pone.0285001.s003.docx]

|  | Male (n=576) | | Female (n=721) | |
| --- | --- | --- | --- | --- |
| Dimension | Mean | (*SD*) | Mean | (*SD*) |
| Total | 2.85 | (0.61) | 2.88 | (0.54) |
| Accessing in healthcare | 3.01 | (0.85) | 3.02 | (0.77) |
| Understanding in healthcare | 3.07 | (0.74) | 3.15 | (0.64) |
| Appraising in healthcare | 2.68 | (0.77) | 2.70 | (0.71) |
| Applying in healthcare | 2.81 | (0.80) | 2.84 | (0.74) |
| Accessing in disease prevention | 2.57 | (0.83) | 2.66 | (0.78) |
| Understanding in disease prevention | 2.92 | (0.82) | 2.96 | (0.71) |
| Appraising in disease prevention | 2.60 | (0.85) | 2.59 | (0.78) |
| Applying in disease prevention | 3.23 | (0.69) | 3.18 | (0.66) |
| Accessing in health promotion | 2.65 | (0.85) | 2.74 | (0.77) |
| Understanding in health promotion | 2.87 | (0.78) | 2.85 | (0.74) |
| Appraising in health promotion | 2.86 | (0.80) | 2.87 | (0.77) |
| Applying in health promotion | 3.11 | (0.79) | 3.13 | (0.73) |

**S3 Table. Descriptive statistics of the 12 dimensions for the AAPHL instrument for sex-stratified subgroup**
