# Supplementary material for: Psychometric properties of novel instrument for evaluating ambient air pollution health literacy in adults
Source: PLoS One. 2023 Jun 16;18(6):e0285001. doi: 10.1371/journal.pone.0285001 (PMC10275446; doi:10.1371/journal.pone.0285001)
Supplement: S4 Table — (DOCX) [file pone.0285001.s004.docx]

**S4 Table. Descriptive statistics of the 12 dimensions for the AAPHL instrument for age subgroup**

|  | Aged 20-64 (n=1157) | | Aged 65 or over (n=140) | |
| --- | --- | --- | --- | --- |
| Dimension | Mean | (*SD*) | Mean | (*SD*) |
| Total | 2.86 | (0.58) | 2.94 | (0.54) |
| Accessing in healthcare | 3.04 | (0.81) | 2.81 | (0.80) |
| Understanding in healthcare | 3.13 | (0.69) | 3.00 | (0.67) |
| Appraising in healthcare | 2.68 | (0.75) | 2.84 | (0.66) |
| Applying in healthcare | 2.82 | (0.77) | 2.85 | (0.72) |
| Accessing in disease prevention | 2.63 | (0.80) | 2.47 | (0.83) |
| Understanding in disease prevention | 2.95 | (0.76) | 2.89 | (0.73) |
| Appraising in disease prevention | 2.58 | (0.81) | 2.68 | (0.83) |
| Applying in disease prevention | 3.21 | (0.67) | 3.20 | (0.68) |
| Accessing in health promotion | 2.72 | (0.80) | 2.50 | (0.88) |
| Understanding in health promotion | 2.86 | (0.76) | 2.81 | (0.75) |
| Appraising in health promotion | 2.88 | (0.78) | 2.69 | (0.84) |
| Applying in health promotion | 3.11 | (0.76) | 3.23 | (0.72) |
